# The ‘ins’ and ‘outs’ of interpregnancy interval effects: Who contributes, and does it matter?

**DOI:** 10.1111/ppe.12969

**Published:** 2023-03-15

**Authors:** Annette K. Regan, Gavin Pereira

**Affiliations:** ^1^ School of Nursing and Health Professionals University of San Francisco San Francisco California USA; ^2^ Fielding School of Public Health University of California Los Angeles Los Angeles California USA; ^3^ Curtin School of Population Health Curtin University Perth Western Australia Australia; ^4^ enAble Institute Curtin University Bentley Western Australia Australia; ^5^ Centre for Fertility and Health (CeFH), Norwegian Institute of Public Health Oslo Norway

Interpregnancy interval (IPI), or the time between the end of one pregnancy and the conception of the next pregnancy, has been associated with an increased risk of adverse pregnancy outcomes. In high‐income settings, short intervals, typically defined as <6 months, have been linked with a ≥20% increase in the odds of preterm birth compared to intervals 18–24 months.^1^ Over the past two decades, the literature evaluating the health effects of IPIs has rapidly increased—as has the debate over whether short intervals are causally related to the risk of preterm birth. Several studies adjusting for a range of confounders or applying sibship study designs that control for time‐invariant confounders (i.e., genetics, race/ethnicity) have either identified insufficient evidence for an association between IPI and preterm birth—or identified much smaller associations compared to other study designs.^2^

Despite their potential contributions, sibship studies of IPI have received criticism for several reasons, one of which is the exclusion of *noninformative* strata from conditional models.^3^ The most influential sibships that contribute to effect estimates are those with discordant outcomes and exposures,^3^ and these individuals have potentially different baseline risks of delivering preterm compared to those who are *ineligible* and, therefore, excluded from sibship analyses.

Klebanoff and Hade^4^ used the National Survey of Family Growth (NSFG) from 2006 to 2015 to evaluate the influence of two ineligibility constraints commonly made in the IPI literature: (i) sample exclusions made in the analysis of sibship studies; and (ii) exclusions of early pregnancy losses commonly made through the use of registry‐based data collections. Ultimately, the authors attempted to answer the question as to whether results from within‐sibship analyses differ from between‐sibship analyses when applied to the same population.

The answer appears to be, ‘*No*.’ The authors apply several good practice recommendations in their analysis, including the presentation of a directed acyclic graph (DAG) to outline their conceptual framework and fine categorisation of IPI categories with 18–23 months as the referent group.^3^ Their models adjust for confounders uniquely available in the NSFG dataset and that may be unshared by siblings (i.e., essential to sibship studies),^5^ including breastfeeding and pregnancy intention. Pregnancy intention has been identified as an influential confounder.^6^ Because the majority of previous studies have relied on registry data and other population‐based sources of information,^1^ they have not been able to incorporate these confounders in their analyses. Importantly, Klebanoff and Hade show that after adjustment for pregnancy intention and breastfeeding, the association between IPI and preterm birth is minimal.

They also modelled the risk of preterm birth using Poisson and conditional Poisson regression for between‐person and within‐person (sibship births) comparisons. Application of the conditional Poisson model is appropriate but differs from conditional logistic regression. The Poisson model conditions the total event count within each stratum, thereby including birthing individuals with one or two preterm births. The conditional logistic regression alternative restricts to discordant strata, thereby including birthing individuals with exactly one preterm birth. To our knowledge, no studies have yet examined the extent to which results differ by the choice of modelling strategy.

As expected, the within‐sibship analysis included only 37% of the total pregnancies during the study period. Reassuringly, the within‐ and between‐person analyses applied to this cohort produced similar results. Moreover, the between‐person analysis applied to this cohort and those not included because they were ineligible, also produced similar results. Despite this reassurance, one stand‐out finding is the modest protective association observed between preterm birth and short intervals <6 months (as compared to intervals 18–23 months for both between and within‐person analyses; see Table 5, Klebanoff & Hade)^4^—which contrasts the existing IPI literature and previous sibship studies^3^ and is a reversal to the harmful associations reported for preterm birth and intervals <6 months (see Tables 1, 3, Klebanoff & Hade).^4^ In fact, even for analyses restricted to stillbirths and live births (see Table 2, Klebanoff & Hade),^4^ which is meant to emulate a registry‐based approach, the authors do not observe any increase in the risk of preterm birth associated with short intervals <6 months—even in an unadjusted model. This does not align with previous register‐based studies, which have identified a 20%–80% increase in the rate of preterm birth associated with IPI <6 months.^1^

This raises the question as to why results from analyses of NSFG are different to findings from previous IPI studies. One explanation is the choice of model. Another is the potential difference in sample characteristics. The NSFG is a national US survey, and in contrast to other IPI studies, is not a population‐based data source. The response rate for the NSFG is approximately 70% among eligible respondents.^7^ Although the NSFG employs a two‐stage sampling strategy to reduce the influence of nonresponse bias, respondents may differ from the general population. A third potential explanation is the influence of misclassification or uncontrolled nonshared confounders.^5^ Registry‐based studies draw from high‐quality clinical information, but often cannot include pregnancies that result in early pregnancy loss. However, the association between short IPI and preterm birth does not appear to be strongly influenced by this misclassification.^8^ In contrast, estimation of IPI from NSFG data relies on accurate recall and self‐report of date of birth and gestational age at delivery. The accuracy of self‐reported gestational age could be reasonably questioned. A recent prospective cohort study suggested that 98% of maternal participants provide accurate gestational age information within 1 week of the best clinical estimate.^9^ However, greater parity and longer time to pregnancy were associated with the less accurate recall of gestational age,^9^ both of which would be problematic for IPI research.

Regardless, the current study provides some reassurance that results from sibship studies align with the results of analyses for those who are ineligible for inclusion in sibship studies. This may suggest that findings are generalizable from sibship studies. There is, unfortunately, no one perfect study, sibship or otherwise, to answer the question, ‘how long should we wait?’ To answer this question, creative study designs and a better understanding of the results from different approaches, as Klebanoff and Hade have provided here, will be needed. The evidence appears to be converging to indicate that if a causal relationship between IPI and preterm birth exists, it is likely much smaller than current global pregnancy spacing guidelines suggest. Indeed, the current global guidelines for pregnancy spacing after pregnancy loss appear to conflict with findings from contemporary studies.^10^ This raises the question of whether existing guidelines are too conservative and should better reflect recent and emerging evidence.

## ABOUT THE AUTHORS

**Annette Regan** is an assistant professor in the School of Nursing and Health Professions at the University of San Francisco where she teaches epidemiology and biostatistics. She is a perinatal epidemiologist and her research focuses on the real‐world evaluation of maternal and child health issues, including birth spacing, vaccines, and maternal and childhood infections.

**Gavin Pereira** is a professor of epidemiology and biostatistics at the Curtin School of Population Health, Curtin University, Australia. He is a current Australian National Health and Medical Research Council research fellow (EL2). He leads an international program of research to better understand, and thereby influence, the biological, social, and environmental determinants of health and well‐being.

## AUTHOR CONTRIBUTIONS

AKR and GP contributed equally to the conceptualization of the article. AKR led the writing of the initial draft and both authors contributed to revisions of the manuscript.

## Data Availability

There are no data associated with this manuscript.
